# Design and Comparative Evaluation of Vancomycin HCl-Loaded Rosin-Based In Situ Forming Gel and Microparticles

**DOI:** 10.3390/gels8040231

**Published:** 2022-04-08

**Authors:** Tiraniti Chuenbarn, Jitnapa Sirirak, Sarun Tuntarawongsa, Siriporn Okonogi, Thawatchai Phaechamud

**Affiliations:** 1 Department of Pharmaceutical Technology, Faculty of Pharmacy, Silpakorn University, Nakhon Pathom 73000, Thailand; chuenbarn_t@su.ac.th; 2 Department of Chemistry, Faculty of Science, Silpakorn University, Nakhon Pathom 73000, Thailand; sirirak_j@silpakorn.edu; 3 Pharmaceutical Intellectual Center “Prachote Plengwittaya”, Faculty of Pharmacy, Silpakorn University, Nakhon Pathom 73000, Thailand; tuntarawongsa_s@su.ac.th; 4 Natural Bioactive and Material for Health Promotion and Drug Delivery System Group (NBM), Faculty of Pharmacy, Silpakorn University, Nakhon Pathom 73000, Thailand; 5 Research Center of Pharmaceutical Nanotechnology, Faculty of Pharmacy, Chiang Mai University, Chiang Mai 50200, Thailand; okng2000@gmail.com; 6 Department of Pharmaceutical Sciences, Faculty of Pharmacy, Chiang Mai University, Chiang Mai 50200, Thailand

**Keywords:** in-situ forming gel, in-situ forming microparticle, rosin, solvent exchange, periodontitis

## Abstract

Vancomycin hydrochloride (HCl) is a glycopeptide antibiotic used to treat serious or life-threatening infections, and it reduces plaque scores and gingivitis in periodontal patients. In this study, vancomycin HCl was incorporated into rosin in situ forming gel (ISG) and rosin in situ forming microparticles (ISM) to generate a local drug delivery system to treat periodontal disease. The physical properties of the ISG and ISM were measured, including pH, viscosity, injectability, adhesion properties, in-vitro transformation, and drug release. Moreover, the effectiveness of antimicrobial activity was tested using the agar-cup diffusion method against *Staphylococcus aureus*, *Streptococcus mutans*, *Porphyromonas gingivalis*, and *Escherichia coli*. Vancomycin HCl-loaded rosin-based ISG and ISM had a pH value in the range of 5.02–6.48 and exhibited the ease of injection with an injection force of less than 20 N. Additionally, the lubricity effect of the external oil phase of ISM promoted less work of injection than ISG and 40–60% rosin-based ISM showed good emulsion stability. The droplet size of emulsions containing 40%, 50%, and 60% rosin was 98.48 ± 16.11, 125.55 ± 4.75, and 137.80 ± 16.8 µm, respectively. Their obtained microparticles were significantly smaller in diameter, 78.63 ± 12.97, 93.81 ± 10.53, and 118.32 ± 15.61 µm, respectively, because the particles shrank due to the solvent loss from solvent exchange. Moreover, increasing the concentration of rosin increased the size of microparticles. After phase transformation, all formulations had better plasticity properties than elasticity; therefore, they could easily adapt to the specific shape of a patient’s gum cavity. Both developed ISG and ISM presented inhibition zones against *S. mutans* and *P. gingivalis*, with ISG presenting significantly more effectively against these two microbes (*p* < 0.05). The vancomycin HCl-loaded rosin ISG and ISM delayed drug release for 7 days with efficient antimicrobial activities; thus, they exhibit potential as the drug delivery systems for periodontitis treatment.

## 1. Introduction

Rosin contains abietic acid as the main structure and a small number of nonacidic compounds [1]. Rosin shows good film-forming and coating properties for enteric-coated drug delivery systems [1,2]. As a natural material, rosin provides biodegradable properties in vivo, as well as non-toxic and biocompatibility features [2]. Furthermore, abietic acid has antibacterial activity against Gram-positive bacteria, such as *Staphylococcus* *aureus* and *Staphylococcus epidermidis*, and provides some effectiveness against resistant *streptococci* and *enterococci*. However, it has no antibacterial effects against Gram-negative bacteria [3]. Rosin exhibits good encapsulating properties for controlled-release drug delivery of tablets; therefore, various active compounds can be incorporated [2].

An in situ forming gel (ISG) is a liquid polymer formulation that transforms into a solid or gel when it comes in contact with biological fluid via solvent exchange and thermal or pH induction [4,5]. This system contains biodegradable polymers, including drugs dissolved in an organic solvent such as *N*-methyl pyrrolidone (NMP), dimethyl sulfoxide (DMSO), 2-pyrrolidone (PYR), or ethyl acetate [6,7]. After injection into a target site, the phase separation caused by the surrounding water leads to transformation of the polymeric solution into gel or matrix-like, which entraps the drug inside as a depot. The ISG reduces the antibiotic drug dose required at the periodontal pocket and is easily accessible to administer the drug to this target site for periodontitis treatment. Borneol, saturated fatty acid, bleached shellac and some natural resins have been employed as the gelling agents of solvent exchanged-induced in situ forming systems [8,9,10,11,12]. However, drug release from an ISG is often initial burst release from rapid drug diffusion via solvent movement outward [6,7]. To reduce the unpredictable release pattern, in situ forming microparticles (ISM) have been developed using the basic knowledge of non-aqueous in oil emulsions. ISM consists of non-aqueous droplets with a drug dissolved in a polymeric solution dispersed in an external oil phase. After contact with physiological fluid, the internal phase of an emulsion solidifies and forms microparticles. ISM emulsions are prepared by using the high shear forces of probe sonication to generate a ready-to-inject formulation or by using the two-syringe connector method to prepare prior-to-inject formulations using a back-and-forth movement of the syringe plunger [7,13]. Due to the presence of the external oil phase, ISM has advantages over the highly viscous ISG, as they reduce the initial burst release and the myotoxicity of the solvent. Moreover, ISM are easily injected through a needle and prolong the drug release [13,14]. However, ISM are less stable owing to the high surface free energy of emulsion droplets. To stabilize the formulation, Voigt et al., 2012 used some emulsifiers to improve the emulsion stability of the poly (D,L-lactide-co-glycolide) (PLGA) solution dispersed in vegetable oil. Glyceryl monosterate (GMS) has been claimed to generate a birefringent layer at the interface between the PLGA droplets and the oil phase. No phase separation occurred in over an hour, while other emulsifiers did not inhibit the phase separation [15]. Bleached shellac-based ISM has been developed for periodontitis treatment using GMS as an emulsifier and olive oil as the dispersing medium [8]. Owing to its enteric properties and aqueous insolubility like bleached shellac, rosin is of interest for controlled drug delivery systems. The biocompatibility and degradable character of rosin are interesting for using as the gelling agent or matrix forming agent of ISG and ISM, respectively. In addition, comparative evaluations of rosin-based ISG and ISM have not been reported previously.

Vancomycin HCl is a glycopeptide antibiotic used to treat serious or life-threatening organisms that are resistant to penicillin, such as methicillin-resistant *Staphylococcus aureus* and multidrug-resistant *Streptococcus epidermidis*. This drug has serious side effects when administered systemically. However, no adverse effects have been reported when vancomycin HCl is used topically [16,17]. It has been reported that 1% vancomycin oral paste applied to periodontal patients reduces the plaque score and gingivitis [18]. Therefore, the vancomycin HCl-loaded rosin ISG and ISM are interesting for comparison of their physicochemical properties, drug release behavior and antimicrobial activities for periodontitis treatment.

In this study, vancomycin HCl was incorporated into rosin-based ISG and ISM formulations to develop local drug delivery systems for treatment of periodontitis against *Staphylococcus aureus*, *Streptococcus mutans*, *Escherichia coli* and *Porphyromonas gingivalis*. The physical properties of the ISG and ISM were investigated, including pH, viscosity, rheology, injectability, adhesion properties, in vitro transformation and drug release behavior and kinetics.

## 2. Materials and Methods

### 2.1. Materials

Rosin was received from Karnchanapon Co., Ltd., Nakhon Pathom, Thailand. Vancomycin HCl (V) (lot no. WXBB5169V, Sigma-Aldrich Co., St. Louis, MO, USA) was used as the model drug. Olive oil (Bertolli^®^, Florence, Italy) was used as the external oil phase for the ISM, and dimethylsulfoxide (DMSO) (lot no. 453035, Fluka, Basel, Switzerland) was used as the solvent. Sheep blood agar (Ministry of public health, Mueang, Nonthaburi District, Thailand), tryptic soy agar and tryptic soy broth (Difco™, Detroit, MI, USA) were used as media for antimicrobial investigations. Glyceryl monostearate (GMS) (type: self-emulsifying grade) (PC Drug, Bangkok, Thailand) was used as an emulsion stabilizer. Potassium dihydrogen orthophosphate (lot no. E23W60, Ajax Finechem, Wollongong, NSW, Australia) and sodium hydroxide (lot no. AF310204, Ajax Finechem, Wollongong, NSW, Australia) were used as components in the phosphate-buffered saline (PBS). Sodium fluorescence (lot no. MKCG7851, Sigma-Aldrich) and agarose (lot no. C7031-17, Vivantia Inc., Emeryville, CA, USA) were used for the microscopic transformation study. *S. aureus* ATCC 25923, *E. coli* ATCC 25922, *S. mutans* ATCC 25175, and *P. gingivalis* ATCC 33277 from Ministry of public health, Mueang, Nonthaburi District, Thailand were used as test bacteria.

### 2.2. Preparation of the Formulations

#### 2.2.1. Preparation of In-Situ Forming Gel (ISG)

The ISG formulations were prepared by dissolving 20, 30, 40, 50, and 60% (*w*/*w*) rosin in DMSO, and the mixture in a closed glass bottle was rotated for 48 h or until a clear solution was obtained using a roller tube mixer (Scilogex MX-T6-S analog tube roller, Scilogex, Rocky Hill, CT, USA). Subsequently, 1% (*w*/*w*) vancomycin HCl was incorporated in rosin solutions. The composition of the formulations are presented in Table 1.

#### 2.2.2. Preparation of In Situ Forming Microparticles (ISM)

The internal phase of the ISM emulsion systems were prepared from the above drug-loaded ISG. GMS (7.5%, *w*/*w*) was incorporated into olive oil by mixing with heating at 60 °C using a magnetic stirrer until a clear solution was obtained to generate the external oil phase. The two phases were merged at a 1:1 ratio using a 3 mL disposable syringe connected with a butterfly Lure-Lock at a rate of 2 cycles/s for 60 s. This preparation technique has been reported previously for ISM preparation [13,15,19]. The obtained concentration of drug and rosin in ISM was half of ISG since the internal phase was diluted with an equal amount of the external phase from the preparation procedure as described previously. The components of these vancomycin HCl-loaded ISM systems are shown in Table 1.

### 2.3. Evaluation of the ISG and ISM Systems

#### 2.3.1. Appearance and Phase Separation of ISM Emulsion

The physical appearances of prepared vancomycin HCl-loaded rosin-based ISG and ISM such as color and transparency were observed. Phase separation of the prepared ISM was investigated in a disposable syringe at room temperature. A 1 mL syringe was filled with the 0.8 mL ISM formula and the syringe was placed in a vertical position and the volume of oil separation was recorded during 1 h.

#### 2.3.2. pH Measurement

The pH values of the pure solvents used in the ISG and ISM formulations were determined using a pH meter (Ultra basic UB-10, Denver Instruments, Bohemia, NY, USA) (*n* = 3).

#### 2.3.3. Viscosity and Rheology Behavior

The viscosity and rheology of the prepared ISG and ISM were investigated using a Brookfield DV-III Ultra programmable rheometer (Brookfield Engineering Laboratories Inc., Middleborough, MA, USA) with spindles (CP40 and CP52) (*n* = 3). Viscosity was measured at room temperature, and the viscosity parameters were determined at different shear rates with a 15 s equilibration time for each shear rate determination (*n* = 3).

#### 2.3.4. Injectability Test

The ISG and ISM were evaluated using a texture analyzer (TA.XT plus, Stable Micro Systems, Godalming, UK) (*n* = 3) in compression mode. A 1 mL syringe was filled with formula and fixed to a stand. The syringe plunger was pressed with the flat face of a cylindrical probe at a constant speed of 1.0 mm·s^−1^ and a constant force of 0.1 N to expel the formulation through a 27-gauge needle with a 20 mm barrel length at room temperature (*n* = 3). The maximum force (N) was selected for analysis, and the area under the curve (AUC) was used to determine the work of expulsion.

#### 2.3.5. Adhesion Properties

An agarose gel was prepared by dissolving 0.6% *w*/*w* agarose powder in PBS with heat until a clear solution was obtained. A 6-cm plastic petri dish was filled with the agarose solution and the gel was allowed to set up. A well was added to the center of the plate using a cylindrical stainless cup to simulate a human periodontal pocket. A 200 μL aliquot of prepared ISG or ISM was used to fill the well and allowed to transform completely into a solid for 1 week. The transformed formulations were evaluated using a texture analyzer (TA.XT plus, Stable Micro Systems, Godalming, UK) in compression mode. The spherical probe moved downward at a constant speed of 0.5 mm/s and penetrated the transformed formulation. The applied force and displacement of the probe were determined as a function of time. The position, at a penetration depth of 2 mm, was held for 60 s. Then, the probe was driven upwards at 10 mm/s. The maximum deformation force (Fmax deformation) was measured as the maximum force when the probe penetrated the transformed formulation. The force after a 60 s hold time was the remaining force (Fremaining) and the adhesion force was the maximum force measured during upward movement of the probe, which was in the negative direction of the force (Fadhesion). The F_remaining_/F_max_ deformation ratio was used as a measure of elasticity/plasticity as previously mentioned [20].

#### 2.3.6. In-Vitro Gel and Microparticle Transformation

A 1 mL disposable syringe was filled with the prepared ISG or ISM and injected through a 27-gauge needle into a test tube containing 6 mL of PBS. The formulations were observed visually. ISM was investigated under an inverted microscope (TE-2000U, Nikon, Kaw, Japan) at magnification of 100×. PBS (pH 6.8) was dropped on the center of a glass slide, a 50 μL drop of the formulation was placed beside the PBS, and the drops were allowed to contact each other. The transformation was observed for 0, 10, 30, 60, and 180 s. The ISM size was measured using an image analyzer program (JMicroVision 1.2.7) (*n* = 75).

An agarose gel was prepared using 0.6% (*w*/*w*) agarose in PBS 6.8 solution. Sodium fluorescence (0.003%, *w*/*w*) was added for color and stirred with heat until a clear light-green colored solution was obtained. The agarose solution was coated on glass slides and cooled until it formed a gel to simulate the human gum cavity. A 50 μL aliquot of the prepared ISG and ISM was dropped beside the gel, and transformation was observed for 0, 10, 30, 60, and 180 s under an inverted microscope (TE-2000U, Nikon, Kaw, Japan) at a magnification of 100×.

#### 2.3.7. In-Vitro Drug Release

Drug release was evaluated using the dialysis method to determine the amount of antibiotic drug released from the ISG and ISM in PBS by filling a dialysis bag with 1 g. The formulation in the dialysis bag (Spectra/Por^®^ membrane MWCO: 6000–8000, lot no. 9200006, Spectrum Laboratory, Inc., Fluka, Switzerland) was placed in a 100 mL PBS bottle. The bottle was swirled in an incubator (Biocotek, Ningbo, China) at 37 ± 0.5 °C and 25 rpm. A 10 mL aliquot of medium was sampled at different time intervals. Absorbance was measured with a UV-VIS spectrophotometer (Cary 60 UV-Vis, Model G6860A, Agilent, Santa Clara, CA, USA) at 280 nm (*n* = 3) as described previously [10].

#### 2.3.8. Topography

To understand the structures of the ISM and ISG after the drug release test, the released formulations were passed through filter paper and completely dried in a freeze dryer (Triad™ Labconco, Kansas City, MO, USA). The surface and cross-section of the remnants were observed by scanning electron microscopy (SEM) (Tescan mira3, Kohoutovice, Czech Republic) at magnifications of 1000×, 10,000×, and 30,000×.

#### 2.3.9. Antimicrobial Activities

**Figure 1:**
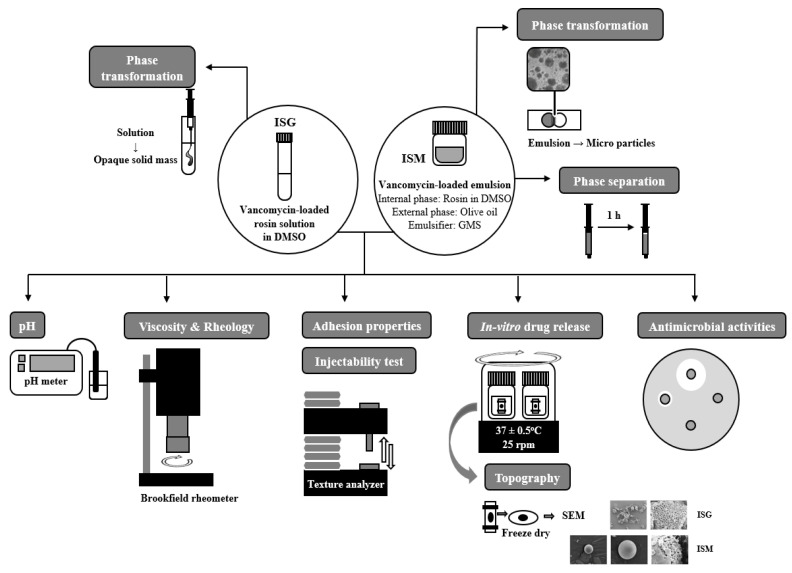
Schematic diagram of the experimental procedure.

The antimicrobial activities of ISG and ISM in the vancomycin HCl -loaded and free-drug formulations were evaluated against standard microbes (*S. aureus* ATCC 6538P and *E. coli* ATCC 25922) and anaerobic microbes (*S. mutans* ATCC 27175 and *P. gingivalis* ATCC 33277) using the agar cup diffusion method. The bacteria inocula were incubated for 36 h in tryptic soy broth and the turbidity of broth suspensions of organisms was checked using 0.5 McFarland standard. Then, the prepared broth suspensions of *S. aureus* ATCC 6538P, *E. coli* ATCC 25922 were swab-spread on the tryptic soy agar plates, whereas sheep blood agar was used as media for antimicrobial testing of *P. gingivalis*. Sterilized cylindrical cups were carefully placed on the surface of the swabbed agar. A 200 μL aliquot of prepared ISG or ISM was added to the cup and incubated at 37 °C for 24 h. The antimicrobial test against anaerobic bacteria was conducted in an anaerobic incubator (Forma Anaerobic System, Thermo Scientific, Waltham, MA, USA) at 37 °C for 72 h. Antimicrobial activities were measured as the diameter (mm) of the inhibition zone (*n* = 3) The schematic diagram representing the procedure of experiment is shown in Figure 1.

### 2.4. Statistical Analysis

All measurements were collected and differences were detected using paired *t*-tests to analyze the size of ISM emulsion and microparticle form using 95% confidence intervals of the differences. Independent *t*-tests were used to analyze the difference between ISG and ISM maximum adhesion force properties using 95% confidence intervals and one-way ANOVA followed by the least significant difference (LSD) post-hoc test or Duncan’s test to analyze the difference in inhibition zones. The significant level was set at *p* < 0.05.

## 3. Results and Discussion

### 3.1. Appearance and Phase Separation

All ISG formulations presented as clear slight yellowish solutions. After emulsification to generate ISM, the phase separation occurred for ISM using 20 and 30% (*w*/*w*) rosin (20RV ISM and 30RV ISM) within 5 min; ISM containing 40% (*w*/*w*) rosin (40RV ISM) had oil separation at 38 min, whereas those comprising 50% and 60% (*w*/*w*) rosin (50RV ISM and 60RV ISM) presented good emulsion stability without phase separation for longer than 60 min. For preparing non-aqueous in oil emulsion of ISM, the suitable emulsion stabilizer or emulsifier is a crucial ingredient; however, the hydrophilic-lipophilic balance typically cannot be normally applied because no aqueous phases present in ISM systems [15]. Practically, glyceryl monostearate (GMS) exhibits as a good emulsifier for various non-aqueous in oil emulsions of ISM owing to its molecular layering formation around the internal droplet surface, protecting the coalescence of the droplet; the high viscous GMS phase surrounding internal emulsion droplets also promotes emulsion stability [8,15,21]. An amount of this emulsifier higher than 5% could stabilize the non-aqueous in oil emulsion of ISM efficiently [22,23]. Increasing the amount in the external phase could efficiently enhance the stability of ISM emulsion against droplet coalescence; nevertheless, the internal phase to external phase ratio of 1:1 exhibited the high amount of drug loading and effective formation into microparticles [21,23,24,25]. The utilization of GMS could stabilize an emulsion of ISM with no signs of phase separation over more than 1 h, independent of whether medium chain triglycerides or sesame oil were employed as a dispersing phase, since a fine amorphous matrix and rod-shape-like GMS crystals are embedded in the dispersing phase of emulsion [15]. Practically, pharmaceutical ISM are in a freshly prepared dosage form; thus, the physical emulsion stability without phase separation for 1 h before administration via injection by the dentist or physician should be acceptable [22,23,25].

### 3.2. pH Measurement

The pH values of the DMSO, ISG and ISM formulations were measured as shown in Table 2. DMSO was alkaline with a pH of 11.20 ± 0.17, whereas ISG and ISM were more acidic (5.02–6.48) owing to the presence of abietic acid of rosin [26,27] and HCl of vancomycin [28,29]. Thus, the pH value decreased after adding increasing amounts of rosin. However, ISM had a slightly higher pH than ISG at the same concentration of rosin, as olive oil has a low acidic value of 0.8 and interferes with the diffusion of vancomycin HCl [30]. In general, vancomycin HCl presented good pH stability of 3.0–5.7 [28,29]. Therefore, the ISG and ISM formulations using rosin as the gelling agent and matrix former, respectively, exhibited appropriate pH for vancomycin HCl.

### 3.3. Viscosity

DMSO showed low viscosity at 3.53 ± 0.08 cps, whereas ISG and ISM indicated increasing viscosity when the amount of rosin was increased (Table 2). The 20–40RV and 40RV ISM formulations exhibited low viscosities (<50 cps) of 7.49 ± 0.60, 16.57 ± 0.84, 30.64 ± 1.02, and 38.62 ± 1.33 cps, respectively. The 50RV and 50RV ISM formulations had medium viscosities (50–100 cps) of 59.98 ± 1.77 and 61.11 ± 1.67 cps, whereas the 60RV and 60RV ISM formulations had high viscosities (>100 cps), (112.45 ± 0.98 and 108.35 ± 2.45 cps), respectively. In comparison, ISM revealed a higher viscosity than ISG in the 40 and 50% rosin formulations because the viscosity of olive oil is rather high at 25 °C [31,32]. However, at the present 60% (*w*/*w*) rosin, ISM showed a slight decrease in viscosity. Moreover, ISM and ISG revealed Newtonian flow as rheological behavior.

### 3.4. Injectability Test

The injectability test was used to explain the expulsion ability of the formulation out of the syringe and needle while pressing the syringe plunger at the injection site [31,32]. In this study, a 27-gauge needle was used as the common needle size for injecting drugs into the gum cavity [33]. The results showed that most of the ISG and ISM formulations had a lower injection force (<10 N), as presented in Table 3, which indicated that they were easy to inject except for 60RV, in which the needle detached during the experiment because of the high friction force between the formulation and the barrel [33,34]. However, when preparing the external phase to generate ISM, 60RV ISM was the easiest to inject with a force of 16.83 ± 1.14 N, as olive oil has a lubricating effect that reduces the friction force [35]. In contrast, the injection forces of the 40RV ISM and 50RV ISM formulations were slightly higher than those of ISG due to the high viscosity of the olive oil, which agrees with the viscosity results. Additionally, the work of injecting the formula also showed a similar trend of more work being required to inject a higher amount of rosin, as presented in Table 3.

### 3.5. Adhesion Properties

A local drug delivery system for treating periodontal disease needs to be inserted into the gum cavity to eliminate bacteria. After insertion, the drug is removed by a chemical reaction and mechanical circulation of the saliva [5,7,36]. An adhesion test was performed to observe attachment between the formulation and the gum [5,6]. The center well of a 0.6% (*w*/*w*) agarose gel represented the gum cavity. After the formulation was injected into the well for 1 week, a spherical cylindrical probe was pressed into the setting formulation, which represented the force from chewing and saliva circulation. As shown in Table 3, the 20RV formulation presented the highest maximum force, owing to the fastest transformation of the formula via a solvent exchange mechanism [8,37]. The hardness of the obtained gels was weakened by lime peel oil addition into doxycycline hyclate-loaded rosin ISG due to the retardation of water diffusion into the system [38]. ISM showed a significant decrease in maximum force because the external phase interfered with the solvent exchange [8]. Thus, the ISM presented a softer appearance than ISG after transformation. Moreover, increasing the rosin concentration reduced the maximum force for ISG and ISM because the hardened rosin, which presented after the transformation process, prevented solvent exchange. Therefore, several parts inside of the high amount rosin formulation were still in emulsion form [8].

ISM presented a higher adhesion force than the ISG at a 95% confidence interval, indicating that ISM should have a better attachment to the gum cavity than ISG, and the 40RV ISM formulation showed the highest force (Table 3). The effect of rosin concentration in ISM showed a similar trend to maximum force because of less solvent exchange. Surprisingly, the adhesion force of the ISG was not different in the 40RV to 60RV formulations. The remaining force after pressing the probe for 60 s revealed that a high concentration of rosin represented the lowest remaining force in both ISG and ISM. After calculating the elasticity properties, all of the formulations had better plasticity properties than elasticity (<1); therefore, they could easily adapt to the specific shape of a patient’s gum cavity [6].

### 3.6. In Vitro Transformation

**Figure 2:**
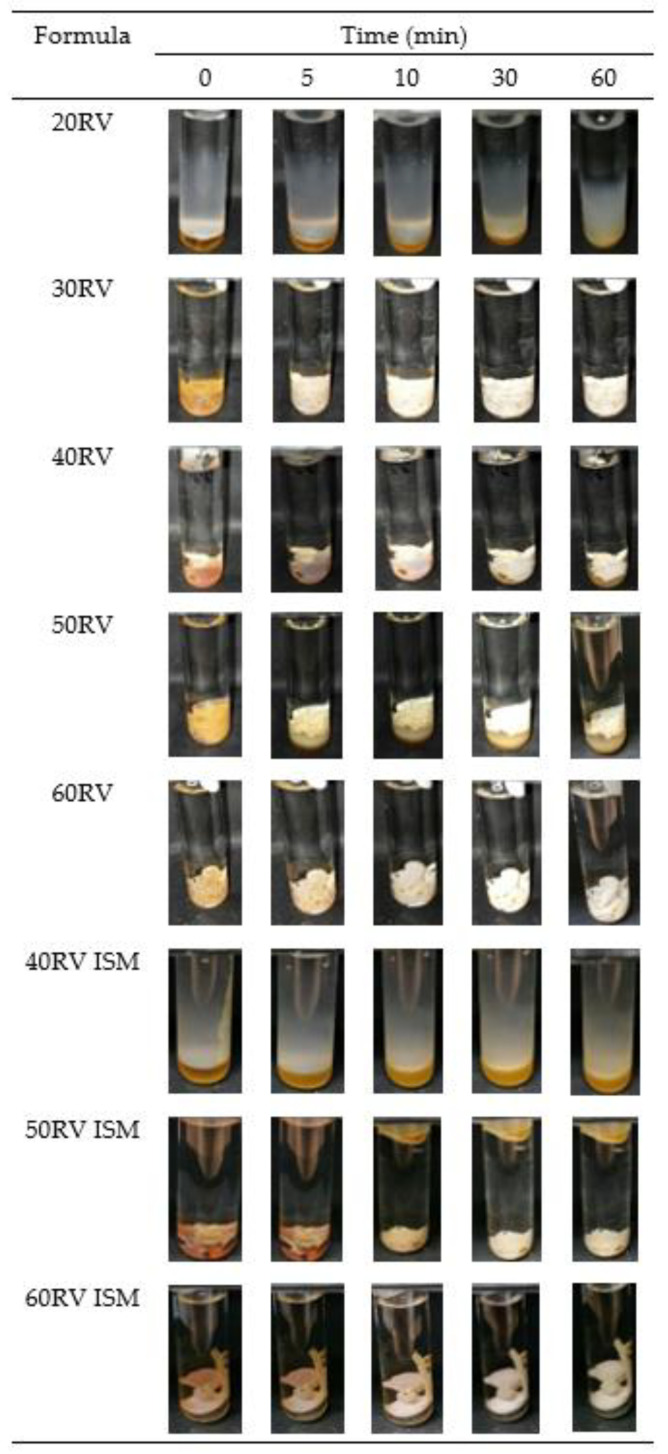
Macroscopic transformation of vancomycin HCl-loaded ISG and ISM containing 20–60% (*w*/*w*) rosin in PBS pH 6.8 at different times.

**Figure 3:**
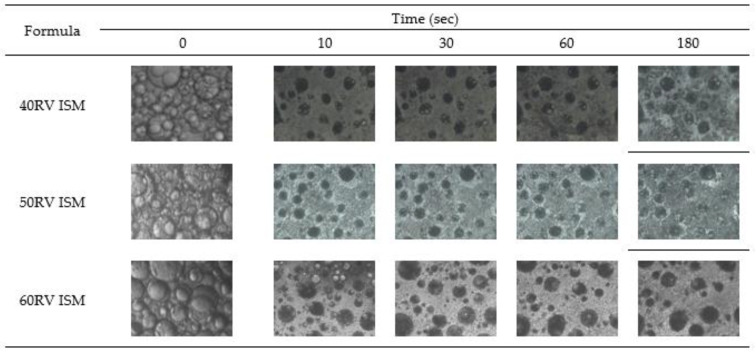
Microscopic transformation of vancomycin HCl-loaded ISM containing 40–60% (*w*/*w*) rosin under the inverted microscope.

**Figure 4:**
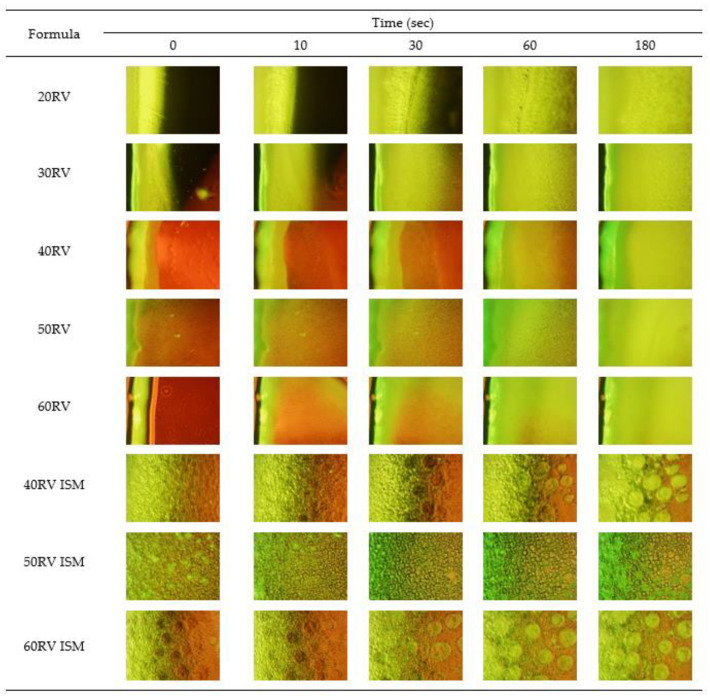
Microscopic transformation of vancomycin HCl-loaded ISG containing 20–60% (*w*/*w*) rosin and ISM containing 40–60% rosin under the inverted microscope using 0.003% *w*/*w* sodium fluorescence in agarose gel as the gum cavity simulation system.

After injecting the formulation into PBS pH 6.8, the surface of all formulations suddenly turned solid and continued to be slowly transformed into an opaque solid mass, which was whiter in color, as illustrated in Figure 2. The surface of the low rosin concentration formulation was less quickly transformed into a solid than the high rosin concentration one, owing to the hydrophobicity of rosin, which precipitates rapidly in water [2]. ISM transformed slower than ISG because the external oil phase interferes with the diffusion of water into the formulation [8,13]. The ISM transformation was performed under an inverted microscope to observe the microscopic transformation, as presented in Figure 3. At the initial time, the formulation was in an emulsion form. After contact with PBS pH 6.8, the emulsion droplets hardened because the rosin precipitated [2,3] and turned into microparticles. The 60RV ISM formulation showed a slightly slower transformation than the others, owing to the high concentration of rosin, which prevented the diffusion of water into the microparticles. To explain the transformation process, 0.003% *w*/*w* sodium fluorescence comprising 0.6% *w*/*w* agarose gel was coated on glass slides to simulate the human gum cavity. The formulation was dropped beside the gel and observed under the inverted microscope. The green color of the PBS diffused into the brown color of the formulation; then, the ISG and ISM were transformed into a solid, as presented in Figure 4. The ISG formulations exhibited phase separation between the DMSO and rosin, as small droplets of DMSO were observed during the transformation process leading to precipitation of rosin [37]. Moreover, the phase separation also occurred in ISM in the internal droplets, which changed color from brown to light green (Figure 4). The PBS diffused inward through the oil phase owing to excess GMS. Then, the PBS attached to the outer shell of the internal droplets because the GMS arranged as a birefringent layer [11] that performed as a barrier for improving the stability of the emulsion. Then, water diffused into the emulsion droplets and the phase separation occurred, leading to the precipitation of rosin microparticles as shown in Figure 4, owing to the aqueous insolubility of this resin. The dissolved vancomycin HCl in the internal phase of that emulsion template could be encapsulated in rosin matrix microparticles. The transformed solid rosin microparticles were observed as a turbid green color.

### 3.7. Size of In Situ Forming Microparticles (ISM)

The diameters of ISM in the emulsion and microparticle forms were measured under the inverted microscope as presented in Table 2. The diameters of both forms were <250 µm, which is the actual size of microparticles [13]. The sizes of the 40RV, 50RV, and 60RV emulsions were 98.48 ± 16.11, 125.55 ± 4.75, and 137.80 ± 16.8 µm, respectively. The microparticles were significantly smaller in diameter in the 40RV ISM, 50RV ISM, and 60RV ISM formations, 78.63 ± 12.97, 93.81 ± 10.53, and 118.32 ± 15.61 µm, respectively, because the particles shrank due to the loss of solvent from the solvent exchange mechanism. Moreover, increasing the concentration of rosin increased the size of microparticles.

### 3.8. Drug Release

**Figure 5:**
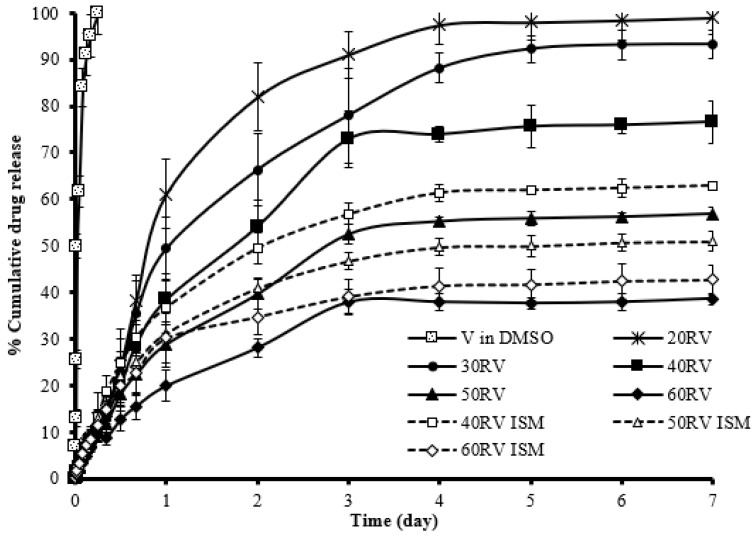
Release of vancomycin HCl from DMSO, ISG, and ISM formulations containing rosin as the gelling agent and the matrix forming agent, respectively, using the dialysis method (*n* = 3).

In this study, drug release was measured using the dialysis method, as illustrated in Figure 5. Vancomycin HCl dissolved in DMSO released completely within 6 h, while both the ISG and ISM formulations retarded vancomycin HCl release with a minimized burst drug release. The commercial product Atridox^®^ containing poly(D,L-lactide) as a matrix material prolongs the drug release for 7 days. This commercial product is injected into the periodontal pocket until the formulation reached the top of the gingival margin, and the amount of filled formulation depends on the individual pocket size of patients [39]. The higher concentration of rosin resulted in greater retardation of drug release from the ISG and ISM, particularly for the systems comprising 60% *w*/*w* rosin. The 40RV ISM and 50RV ISM exhibited a greater sustained ability in drug release than ISG owing to the outer oil phase retarding solvent exchange and drug release. Thus, these results agreed with the phase transformation results. However, there was no significant difference in drug release from the ISG and ISM containing 60% *w*/*w* rosin. The cumulative drug release of the 40–60% *w*/*w* rosin formulations did not reach 100% in either the ISG or ISM because of drug entrapment in the rosin matrix and microparticles during hardening of the rosin matrix. The sustained drug release especially from ISM indicated the gradual diffusion of both drug and solvent; thus, the myotoxicity was potentially minimized. In practice, the myotoxicity was determined in vitro from a cumulative release of creatine kinase (CK) from an isolated rat extensor digitorum longus muscle, whereas an in vivo study assessed the area under the plasma CK-curve after intramuscular injection of male Sprague Dawley rats to evaluate muscle damage [8,40]. The CK level reported previously after injection into an ISM system was significantly minimal; thus, this indicated a lower acute myotoxicity when compared to the injection with polymeric solutions or its solvent [40,41]. With the polymer solutions such as the internal phase, the muscle tissue comes into immediate contact with the solvent after injection, whereas in the ISM system, the external oil phase initially presents as a partial barrier between the muscle tissue and the internal polymeric phase, thus resulting in better biocompatibility and minimized muscle damage [21]. In addition, the ISM systems have a significantly reduced initial burst release as previously described; therefore, the reduced myotoxicity could be achieved.

The low concentration formulations of rosin, including 20RV, 30RV, and 40 RV, presented first-order vancomycin HCl release. In contrast, the 50RV, 60RV, and ISM formulations presented with Higuchi’s release kinetics as shown in Table 4, which means that the rate of drug diffusion from the ISG and ISM was greater than rosin matrix degradation [36]. Moreover, the ISG formulations presented a non-Fickian release mechanism, whereas all of the ISM formulations exhibited the Fickian release mechanism because the rosin relaxation time was much greater than the characteristic solvent diffusion time [41]. These prolongations of drug release with diminishment of burst drug liberation corresponded to the matrix formation of rosin, which is well-known as a controlled release material [42] and the external hydrophobic oil presented as a barrier to solvent diffusion of ISM. The dissolved drug slowly migrates through the rosin structural matrix after it was entrapped in the rosin microparticles. Multi-particulate systems such as ISM minimize variations in implant morphology (after solvent movement) and provide more consistent and reproducible drug release profiles [43]. Moreover, the outer oil phase retards the diffusion of the aqueous phase in which drug diffusion was sustained, which corresponds to the report that clove oil could minimize the burst release of a drug from eudragit RS-based ISG [44].

### 3.9. Surface Topography

**Figure 6:**
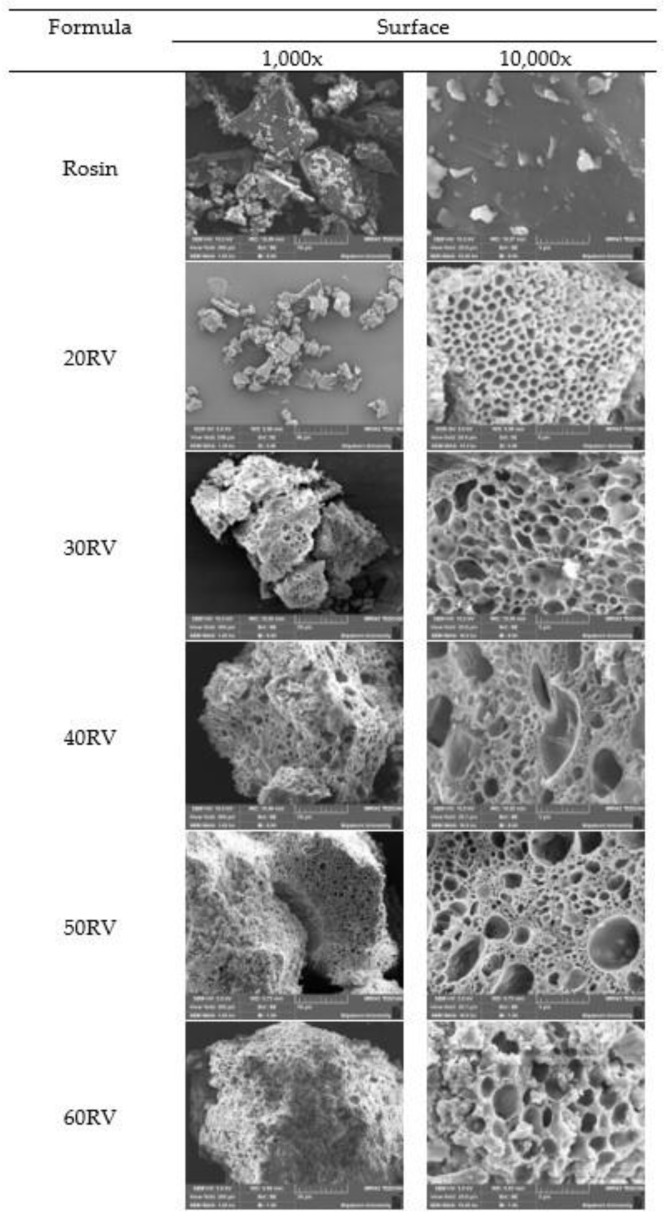
SEM micrographs of the surface of rosin and the vancomycin HCl-loaded ISG formulations at 1000× and 10,000×.

The SEM micrographs of the V-loaded ISG formulations are presented in Figure 6. The rosin had no pores at the surface, while the ISG formulations exhibited a porous structure, indicating that DMSO could change the structure of rosin during transformation due to phase separation. The dynamic computerized modelling indicated the nucleation of fatty acid from phase separation of lauric acid and palmitic acid-based in situ forming matrices using DMSO as a solvent at the initial stage of solvent exchange after their contacting the aqueous phase [45]. Therefore, the porous topography was generated from phase separation of rosin via DMSO dilution with solvent diffusion outward, whereas an aqueous solvent invaded to provoke more rosin phase separation into a sponge-like mass. Increasing the concentration of rosin affected the size and uniformity of the pores. A high concentration of rosin promoted various pore sizes, which exhibited rapid release during the initial phase followed by slower release. The small size of the pores trapped the drug inside; thus, the release profile did not reach 100%.

**Figure 7:**
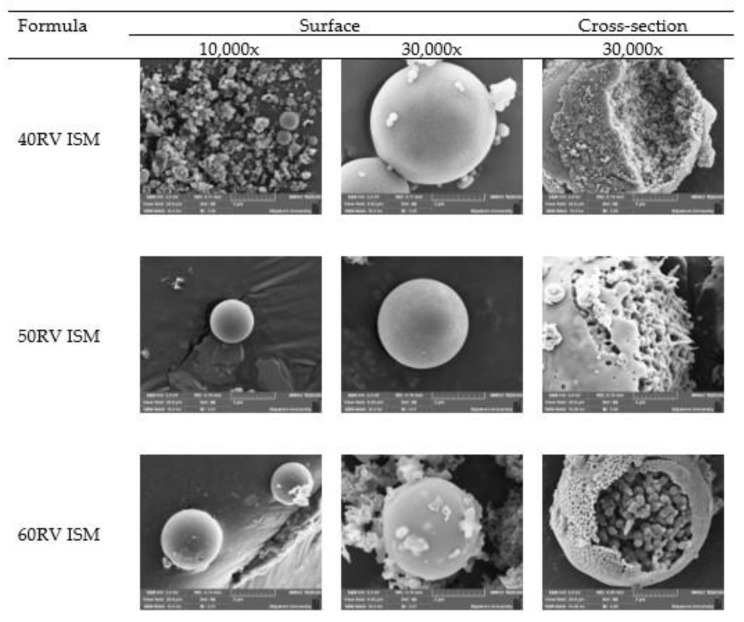
SEM micrographs of the surface and cross-section of the vancomycin HCl-loaded ISM formulations at 10,000× and 30,000×.

The SEM micrographs of the ISM formulation revealed a spherical shape with a smooth surface due to the GMS birefringent layer [11], as shown in Figure 7. The cross-sectional study also revealed the porous structure owing to DMSO diffusion outward. DMSO has been classified as a Class 3 solvent and recommended for utilization less than 50 mg/day [46,47]. Practically, a small volume of the ISG or ISM was administered by the dentist via injection into the periodontal pocket. Consequently, the amount of DMSO is received (about 6 μL) and considered as a very minimal amount, which does not exceed the recommended daily dose, and there is a flow of crevicular fluid to minimize the DMSO concentration at the target site over time [48]. In addition, the safety of DMSO has been explored as a penetration enhancer, which causes a partial local toxicity only applied at high concentrations [49]. In dentistry, DMSO was applied in the oral cavity and exhibited non-irritative effects [50,51]. DMSO showed no cytotoxic effects on the pulp tissue repair-related activity of odontoblast-like cells [52]. Nowadays, the FDA has approved DMSO for treatment of an interstitial cystitis even in pediatric patients and it has been reported as a solvent in in situ forming implants [53]. It has also been employed as a solvent for in situ forming systems comprising fatty acid [45,54], borneol [10] and bleached shellac [8] as the matrix forming agents. DMSO has also been described as a solvent for different dosage forms such as risperidone and paliperidone-loaded in situ forming implants for the treatment of atypical antipsychotics [53]. Therefore, the developed vancomycin HCl-loaded ISG and ISM showed potential as an intra-periodontal drug delivery system, with little or no systemic uptake. Thus, an apparent smaller dose was required for efficient treatment and the side effects could be diminished.

### 3.10. Antimicrobial Activities

**Figure 8:**
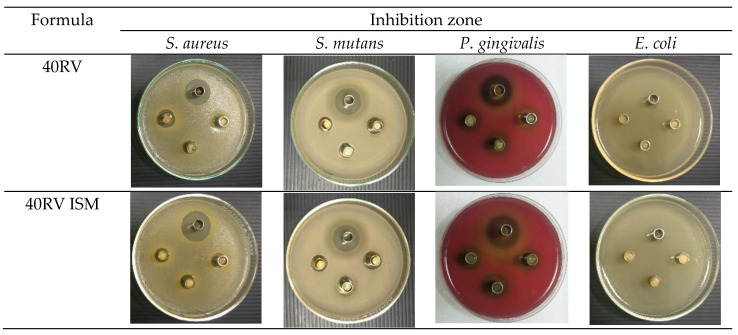
Photographs of the inhibition zone of 40RV and 40RV ISM formulations against *S. aureus*, *S. mutans*, *P. gingivalis*, and *E. coli* (*n* = 3) and the control group (VD) at upper side cup.

Typically, the localized drug delivery formulation has antimicrobial activities against pathogens to treat periodontal disease [55]. In this study, vancomycin HCl-loaded ISG and ISM were tested for their antimicrobial activities against *S. aureus*, *S. mutans*, *P. gingivalis,* and *E. coli* (Table 5). Normal saline solution (NSS) was used as the negative control, which developed no inhibition zones against any of the microbes. DMSO resulted in an inhibition zone against 4 microbes because a high concentration of DMSO leads to cell shrinkage [56]. Vancomycin HCl in DMSO (VD) also presented an inhibition zone, which was more effective than pure DMSO against 4 microbes because DMSO enhances drug transport into bacterial cells [57,58]. Owing to free drug diffusion in rosin-free systems, VD showed an inhibition zone significantly larger than other formulations against *S. mutans* and *P. gingivalis* (*p* < 0.05). Both ISG and ISM showed inhibition zones against *S. mutans* and *P. gingivalis*, with ISG presenting significantly more effectiveness against these two microbes (*p* < 0.05). Increasing the amount of rosin exhibited smaller inhibition zones for inhibiting bacteria growth due to sustainable drug release with greater rosin matrix formation. The ISG also developed an inhibition zone against *S. aureus*, whereas only the 40RV ISM formulation revealed antimicrobial activity. The effective retardation of drug diffusion of high rosin loaded ISM might diminish their inhibition of this microbe; nevertheless, antimicrobial activity against *S. mutans* and *P. gingivalis* remained. Moreover, the ISG formulation with a low concentration of rosin inhibited *E. coli*, while none of the ISM formulations were effective (Table 5). Photographs of the inhibition zones of 40RV and 40RV ISM formulations against *S. aureus, S. mutans, P. gingivalis,* and *E. coli* and the control group (VD) are shown in Figure 8. The addition of lime peel oil into the doxycycline hyclate-loaded rosin ISG modifies drug release rates and promotes the antimicrobial activities [38].

Vancomycin HCl is a glycopeptide antibiotic used to treat serious or life-threatening organisms that are resistant to penicillin such as methicillin-resistant *S. aureus*, and it exhibits higher susceptibility to Gram-positive bacteria than Gram-negative bacteria such as *E. coli* [59,60]. Therefore, the VD and formulations showed rather less inhibition zone against *E. coli* (Table 5). The vancomycin HCl-loaded rosin-based ISM formulation was interesting both for its rapid matrix formation and sustainable drug release with acceptance for injectability. Rosin has been used as main additives in many pharmaceutical dosage forms since it is generally regarded as a nontoxic and nonirritant material [61]. Thus, this resin exhibits the potential use as a safe material for periodontitis treatment. Moreover, rosin is a hydrophobic material; thus, the drug hardly diffused past this resin and the sustainable drug release was achieved. For consideration of antibiotic susceptibility, the minimum inhibitory concentrations (MICs) of vancomycin HCl against *S. aureus*, MRSA, *S. mutans* and *P. gingivalis* are 1–2, 0.125–1, 0.63 and 4 µg/mL, respectively [62,63,64,65]. Typically, a volume of gingival crevicular fluid of 5–20 μL is evident in the periodontal pocket of patients with periodontitis [60]. When 10 μL of formulation was injected into this pocket comprising 10 μL gingival crevicular fluid, the amounts of vancomycin HCl released at 1, 4 and 7 days (as shown in Figure 5) from ISG and ISM were 1.5, 2.5 and 2.65 mg/mL, and 1.65, 2.15 and 2.40 mg/mL, respectively, which were apparently above the MIC against all test microbes. Thus, vancomycin HCl is an antibiotic of interest for periodontitis treatment due to its broad spectrum of efficient antibacterial activities against pathogens involved in periodontitis and MRSA, and it could be the active compound in the near future when there is the incidence of drug resistance in periodontitis pathogens after using other traditional antibiotics. The vancomycin HCl-loaded ISG and ISM are expected to transform into solid-like matrices after contact with crevicular fluid in the periodontal pocket and retard drug release over 7 days. The incorporated molecules and drugs can be controllably released from drug delivery systems such as nanogel and these two developed ISG and ISM systems using various mechanisms including diffusional release, and release driven by polymer degradation or as a response to an external stimulus such as a solvent exchange [66]. Furthermore, these two developed ISG and ISM systems should exhibit efficient antimicrobial activities and perform as a potentially effective localized antibacteria drug delivery system for periodontitis treatment.

## 4. Conclusions

Solvent exchange induced rosin-based ISG and ISM were successfully developed as local drug delivery systems to transform into hardened reservoirs for controlling drug release. Good emulsion stability could be achieved for ISM comprising 40–60% rosin in the internal phase. Vancomycin HCl-loaded rosin ISG and ISM exhibited the ease of injection with an injection force of less than 20 N. ISM exhibited less work of injection than ISG owing to the lubricity effect of the external oil phase. Their phase transformation was attained from solution and emulsion into a gel state and matrix comprising microparticles, respectively, after contact with an aqueous environment. The resin solution and emulsion droplets were hardened because of the phase separation of rosin. The obtained microparticles were significantly smaller in diameter than that of emulsion droplets because of the particle shrinkage from solvent loss during solvent exchange. Increasing the concentration of rosin enlarged the size of ISM. The 40RV ISM presented the highest adhesion force with the plasticity property of being easily adapted to an injection site such as the periodontal pocket. Furthermore, ISM illustrated more retardation of drug release than ISG owing to the presence of an external oil phase. Additionally, both ISG and ISM had antimicrobial activities against *S. mutans* and *P. gingivalis*, indicating the possibility for periodontal pocket drug delivery to increase the effectiveness of periodontitis treatment and reduce drug side effects.

## Figures and Tables

**Table 1:** Composition of vancomycin HCl-loaded rosin-based ISG and ISM formulations.

Formulation	Vancomycin HCl (V)	Rosin (R)	DMSO	Olive Oil	GMS
20RV	1.0	20	79.0	-	-
30RV	1.0	30	69.0	-	-
40RV	1.0	40	59.0	-	-
50RV	1.0	50	49.0	-	-
60RV	1.0	60	39.0	-	-
20RV ISM	0.5	10	39.5	42.5	7.5
30RV ISM	0.5	15	34.5	42.5	7.5
40RV ISM	0.5	20	29.5	42.5	7.5
50RV ISM	0.5	25	24.5	42.5	7.5
60RV ISM	0.5	30	19.5	42.5	7.5
V in DMSO	1.0	-	99.0	-	-

**Table 2:** pH and viscosity of DMSO, ISG, and ISM formulations using rosin as the component (*n* = 3) and sizes of the ISM-emulsion and ISM-microparticle (*n* = 150).

Formula	pH	Viscosity (cPs)	Size (µm)	
			Emulsion	Microparticle
DMSO	11.20 ± 0.17	3.53 ± 0.08	-	-
20RV	5.89 ± 0.05	7.49 ± 0.60	-	-
30RV	5.56 ± 0.06	16.57 ± 0.84	-	-
40RV	5.17 ± 0.09	30.64 ± 1.02	-	-
50RV	5.15 ± 0.08	59.98 ± 1.77	-	-
60RV	5.02 ± 0.14	112.45 ± 0.98	-	-
40RV ISM	6.48 ± 0.06	38.62 ± 1.33	98.48 ± 16.11 *	78.63 ± 12.97 *
50RV ISM	6.46 ± 0.01	61.11 ± 1.67	125.55 ± 4.75 *	93.81 ± 10.53 *
60RV ISM	5.93 ± 0.02	108.35 ± 2.45	137.80 ± 16.84 *	118.32 ± 15.61 *

* Indicates a significant difference at 95% confidence interval between the size of ISM emulsion and microparticle form by using a paired *t*-test analysis.

**Table 3:** Force and AUC of injectability, and adhesion properties of the vancomycin HCl-loaded rosin-based ISG and ISM formulations (*n* = 3).

Formula	Injectability Force (N)	Injectability AUC (N.s)	Adhesion Properties			
			Maximum Force (N)	Adhesion Force (N)	Remaining Force (N)	Elastic Properties
20RV	1.10 ± 0.01	20.59 ± 0.42	3.786 ± 0.172	−0.026 ± 0.010	1.364 ± 0.244	0.36
30RV	2.73 ± 0.12	37.89 ± 2.24	3.579 ± 1.170	−0.002 ± 0.001	2.123 ± 0.773	0.59
40RV	3.10 ± 0.04	56.19 ± 0.57	0.856 ± 0.065 *	−0.050 ± 0.001 *	0.057 ± 0.026	0.07
50RV	7.98 ± 0.17	140.48 ± 5.29	0.737 ± 0.084 *	−0.051 ± 0.001 *	0.052 ± 0.003	0.07
60RV	-	-	0.420 ± 0.043 *	−0.053 ± 0.002 *	0.026 ± 0.005	0.06
40RV ISM	5.36 ± 0.02	92.24 ± 2.58	1.227 ± 0.329 *	−3.4658 ± 0.517 *	1.128 ± 0.350	0.91
50RV ISM	8.31 ± 0.73	135.32 ± 3.75	0.133 ± 0.077 *	−2.010 ± 0.525 *	0.003 ± 0.002	0.03
60RV ISM	16.83 ± 1.14	272.89 ± 17.69	0.060 ± 0.016 *	−0.192 ± 0.019 *	0.005 ± 0.001	0.08

* indicate a significant difference at 95% confidential interval between ISG and ISM maximum and adhesion force in adhesion properties by using independent *t*-test analysis.

**Table 4:** Comparison of the degrees of goodness-of-fit from curve fitting the release profiles of vancomycin HCl released from rosin-based ISG and ISM in PBS pH 6.8 using the dialysis membrane method for the different release models.

Formula (% *w*/*w*)	Zero Order		First Order		Higuchi’s		POWER LAW			Release Mechanism
	cd	msc	cd	msc	cd	msc	cd	msc	N ± S.D.	
V in DMSO	0.7061	0.9135	0.9850	3.9777	0.8905	1.9892	0.9089	2.0846	0.4035 ± 0.075	Fickian
20RV	0.8602	1.7970	0.9899	4.4740	0.9437	2.7598	0.9495	2.8144	0.5763 ± 0.075	Non-Fickian
30RV	0.8991	2.1226	0.9947	5.1257	0.9615	3.1398	0.9727	3.4319	0.5947 ± 0.075	Non-Fickian
40RV	0.8960	2.0923	0.9816	3.8771	0.9615	3.1384	0.9714	3.3842	0.5894 ± 0.075	Non-Fickian
50RV	0.8860	2.0004	0.9235	2.4528	0.9772	3.6630	0.9787	3.6774	0.5408 ± 0.075	Non-Fickian
60RV	0.8710	1.8772	0.8678	1.9058	0.9749	3.5668	0.9742	3.4882	0.5231 ± 0.075	Non-Fickian
40RV ISM	0.8083	1.4811	0.8660	1.8920	0.9572	3.0330	0.9556	2.9443	0.4762 ± 0.075	Fickian
50RV ISM	0.7828	1.3560	0.7715	1.3585	0.9484	2.8475	0.9539	2.9061	0.4415 ± 0.075	Fickian
60RV ISM	0.7383	1.1698	0.6725	0.9987	0.9245	2.4661	0.9413	2.6643	0.4131 ± 0.075	Fickian

**Table 5:** Inhibition zone diameters of NSS, DMSO, vancomycin HCl-loaded rosin-based ISG and ISM formulations against *S. aureus*, *S. mutans*, *P. gingivalis*, and *E. coli* (*n* = 3).

Formula	Inhibition Zone (mm)			
	*S. aureus*	*S. mutans*	*P. gingivalis*	*E. coli*
NSS	-	-	-	-
DMSO	12.67 ± 0.58	11.00 ± 0.00 ^a^	13.67 ± 1.15 ^d^	12.00 ± 0.00
VD	23.67 ± 0.58	22.67 ± 0.58 ^a,b,c^	23.00 ± 0.00 ^d,e,f^	13.67 ± 0.58
20RV	23.33 ± 0.58	22.00 ± 0.00 ^b^	18.33 ± 0.58 ^e^	12.33 ± 0.58
30RV	22.67 ± 1.15	18.67 ± 0.58 ^b^	18.00 ± 0.00 ^e^	11.33 ± 0.58
40RV	14.67 ± 0.58	14.33 ± 0.58 ^b^	14.67 ± 0.58 ^e^	-
50RV	11.67 ± 0.58	11.67 ± 0.58 ^b^	12.67 ± 0.58 ^e^	-
60RV	11.00 ± 0.00	11.00 ± 0.00 ^b^	11.67 ± 0.58 ^e^	-
40RV ISM	10.00 ± 0.00	15.00 ± 0.00 ^c^	13.67 ± 0.58 ^f^	-
50RV ISM	-	13.67 ± 0.58 ^c^	12.00 ± 0.00 ^f^	-
60RV ISM	-	12.67 ± 0.58 ^c^	11.67 ± 0.58 ^f^	-

No inhibition zone; NSS = Normal saline solution; the superscripts ^a–f^ indicate a significant difference (*p* < 0.05) by using one-way ANOVA followed by an LSD post-hoc test.

## Data Availability

The data presented in this study are available on request from the corresponding author.
